# Leaky and waveguide modes in biperiodic holograms

**DOI:** 10.1038/s41598-021-89971-1

**Published:** 2021-05-26

**Authors:** Hamid Keshmiri, Fahimeh Armin, Kareem Elsayad, Frank Schreiber, Mauricio Moreno

**Affiliations:** 1 Institute of Solid State Electronics, Vienna University of Technology, Vienna, Austria; 2 Division of Biodeterioration and Reference Organisms, Federal Institute for Materials Research and Testing, Berlin, Germany; 3 Department of Computer Science, Université du Québec à Montréal, Montreal, Canada; 4 Advanced Microscopy Facility, Vienna Biocenter Core Facilities GmbH, Vienna, Austria; 5 Department of Electronics, University of Barcelona, Barcelona, Spain

**Keywords:** Surface patterning, Sensors, Nanostructures

## Abstract

This study details a theoretical analysis of leaky and waveguide modes in biperiodic all-dielectric holograms. By tuning diffraction orders and subsequently confining local density of optical states at two distinct resonance wavelengths, we present a new class of highly sensitive refractive index biosensing platforms that are capable of resolving 35.5 to 41.3 nm/RIU of spectral shift for two separate biological analytes**.**

## Introduction

Being confined below its cut-off frequency, the concept of leaky modes has been extensively applied in the field of antennas and waveguide propagation at microwave frequencies^1^. Recently the same concept has been transferred into the field of optics, where leaky modes are being exploited in diverse structures including optical filters, antennas and biosensors^2–6^. Leaky wave based biosensors are considered desirable because of their sharp resonances in the reflection spectrum, especially when realized in the context of plasmonic nanopatterns^6,7^. However, such architectures rely on metals for the excitation of surface plasmon modes and therefore limit the efficiency of a sensor from the inherent Ohmic losses of the metal. Nevertheless, metal clad leaky waveguides have previously been reported for biosensing applications e.g. the detection of bacterial spores^8^. We show that as well as being less lossy than their metallic counterparts and having desirable performance parameters, they also exhibit a second equally sharp resonance in their reflectivity spectrum that can be exploited to further improve their surface sensitivity via simultaneous multiplexed detection and fitting. Our work theoretically features the merits of using leaky modes from an all-dielectric relief grating for developing a biosensor.

## Leaky mode in waveguides

Distinct from microwave wavelengths, the optical regime supports leaky waves only by multilayer stacks and periodic structures. This can be understood from the Helmholtz wave equation for which leaky waves, surface waves and lateral waves, are all possible solutions. In the dispersion relation, if *k* is the propagation constant, lateral waves will contribute to the branch cuts, while surface waves are related to real poles, and leaky waves are related to the complex poles in the complex plane of *k* = *k′* + *ik′′* ($i=\sqrt{-1}$ is the unit imaginary number, *i*)^9^. These complex pole singularities will only emerge in the solution of the dispersion relation for multilayer and periodic structures.

Moreover, leaky waves, like surface waves, will have a discrete spectrum because they are related to pole singularities and not branch cuts^10,11^. The power flow (Poynting vector) for leaky waves will generally be at an acute angle relative to structure surface. The modes are called either forward or backward leaky waves depending on whether the in-plane component of the power flow is in the same or opposite orientation as the power flow inside the structure, respectively. Multilayer structures can only support forward leaky waves e.g. in a planar waveguide^12^, while periodic structures or gratings support both forward and backward leaky waves, which for certain applications make them desirable^11^.

## Periodic leaky wave design

Hessel and Oliner were the first to discuss leaky waves in periodic structures and gratings^13^. Although they focused on metallic gratings, the theory applies equally well for all-dielectric structures^9,14^. Because of the periodicity of the grating structure, the scattered energy from the surface of the structure to the medium covering the grating, will leave the surface by an angle *θ*_*m*_, and thus one can write^10^

1 $$\begin{array}{*{20}c} {k\sin \theta_{m} = \beta = \beta_{0} + \frac{2\pi m}{\Lambda }} & {m = 0, \pm 1, \pm 2, \ldots } \\ \end{array}$$

where the left side of the equation is the projection of the wave vector in the propagation plane. Also, *k* is the wave propagation factor in the cover which is equal to *2πn/λ*, with *λ* being the wavelength of light in free space and *n* the refractive index of the cover medium. *β*_0_ is the propagation factor of the wave moving along the grating, *Λ* is the period of the grating, and *β* is the propagation factor along the grating for the *m-th* space harmonic. For the energy to leak out of the grating, *θ*_*m*_ has to be a real angle and thus

2 $$\left| {\frac{k}{\beta }} \right| < 1,$$

for the values of *m* that Eq. (2) is valid, there exists a leaky wave. Forward leaky waves are related to positive values of *m*, while the negative values will lead to the backward leaky waves.

**Figure 1:**
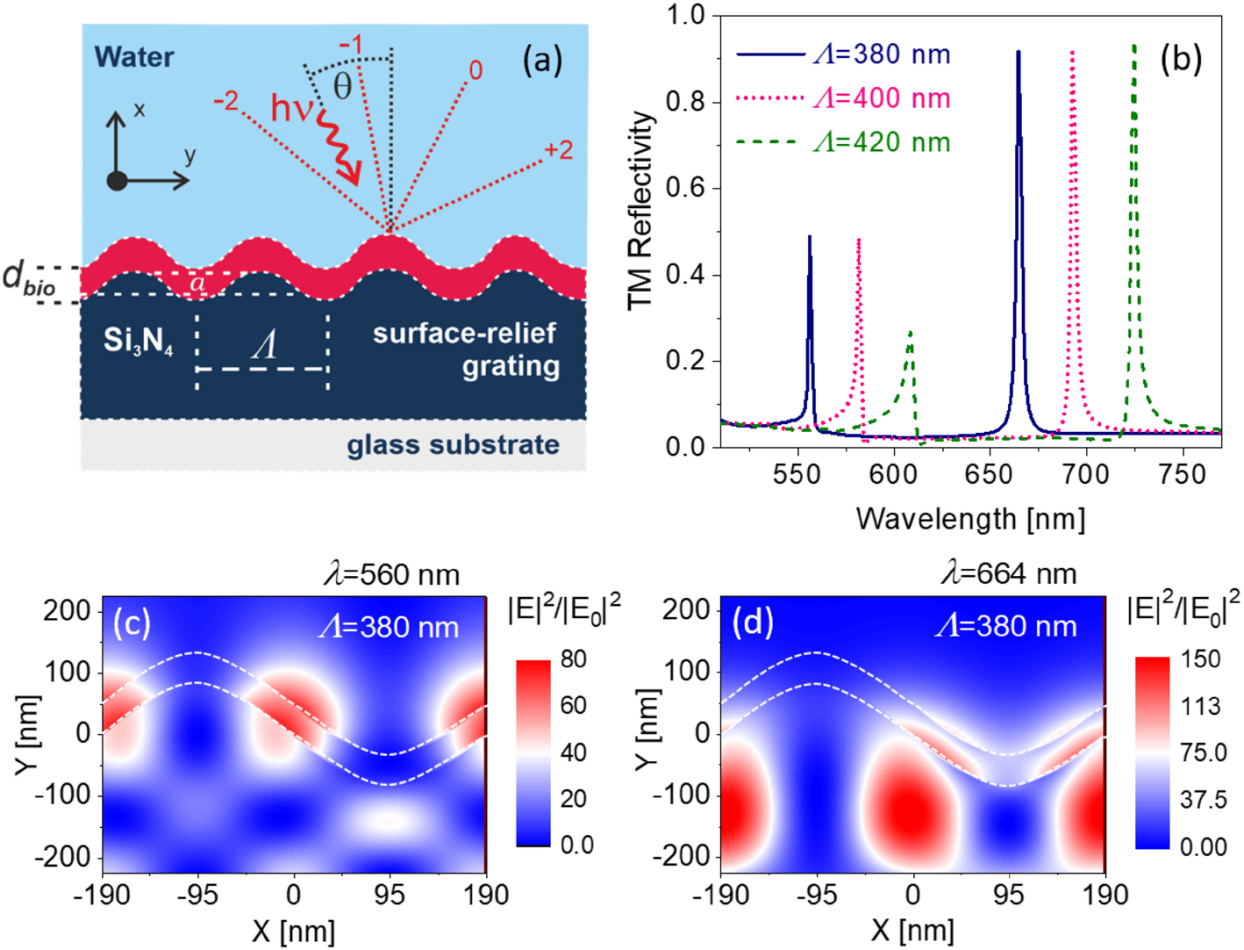
(**a**) Illustration of the leaky wave waveguide-grating. (**b**) TM-polarized reflectivity spectrum as a function of wavelength (*λ*) for single-period waveguide design with different periods (*Λ* = 380 nm, *Λ* = 400 nm, and *Λ* = 420 nm). Also, no biolayer (*d*_*bio*_ = 0) is considered. (**c**,**d**) Normalized electric field intensity profiles above and within such a grating with *Λ* = 380 nm at two different resonant wavelengths. The dotted lines represent the layered of the biological analyte.

As experimentally realized in^15^, the layer structure of the designed leaky waveguides is based on a relief grating made of Si_3_N_4_ assumed on top of a glass substrate and is in contact with an external aqueous medium (illustrated in Fig. 1a). The thickness, *t*, of the waveguide-grating is 200 nm and is covered by a thin layer of a biological analyte with a coating thickness of *d*_*bio*_. In our simulations, it is assumed that an aqueous medium with a refractive index of 1.333 covers the structure surface. For the structure and material used here, the above calculations suggest the periodic hologram will support leaky waves in the wavelength, *λ*, range of 500–650 nm for the periods of the grating, *Λ* between 300 and 450 nm.

The reflectance spectra in the Transverse Magnetic (TM) mode for the gratings with different periods were calculated using the finite-difference time-domain method (Lumerical Inc.) and are shown in Fig. 1b. The results show that the first resonances of the reflectivity spectra occur in the predicted wavelength range (Fig. 1b). In addition to these predictable leaky modes, we observe other resonances, corresponding to distinct waveguide modes, that occur at longer wavelengths in the reflectivity spectrum for each structure.

To gain insight into these higher wavelength resonances, we calculated the normalized electric field profile at the wavelengths of the two resonances for one such structure (Fig. 1c,d). The results show that the second resonance contributes to a waveguide mode in Si_3_N_4_ grating structure. However, as Fig. 1b suggests both resonances can be controlled by the period of the grating, while the grating amplitude would only affect the coupling efficiency and not the resonance wavelengths. Thus, one can envision further tuning of such structures by optimizing the amplitude of the grating and hence the coupling efficiency.

## Biperiodic all-dielectric holograms

**Figure 2:**
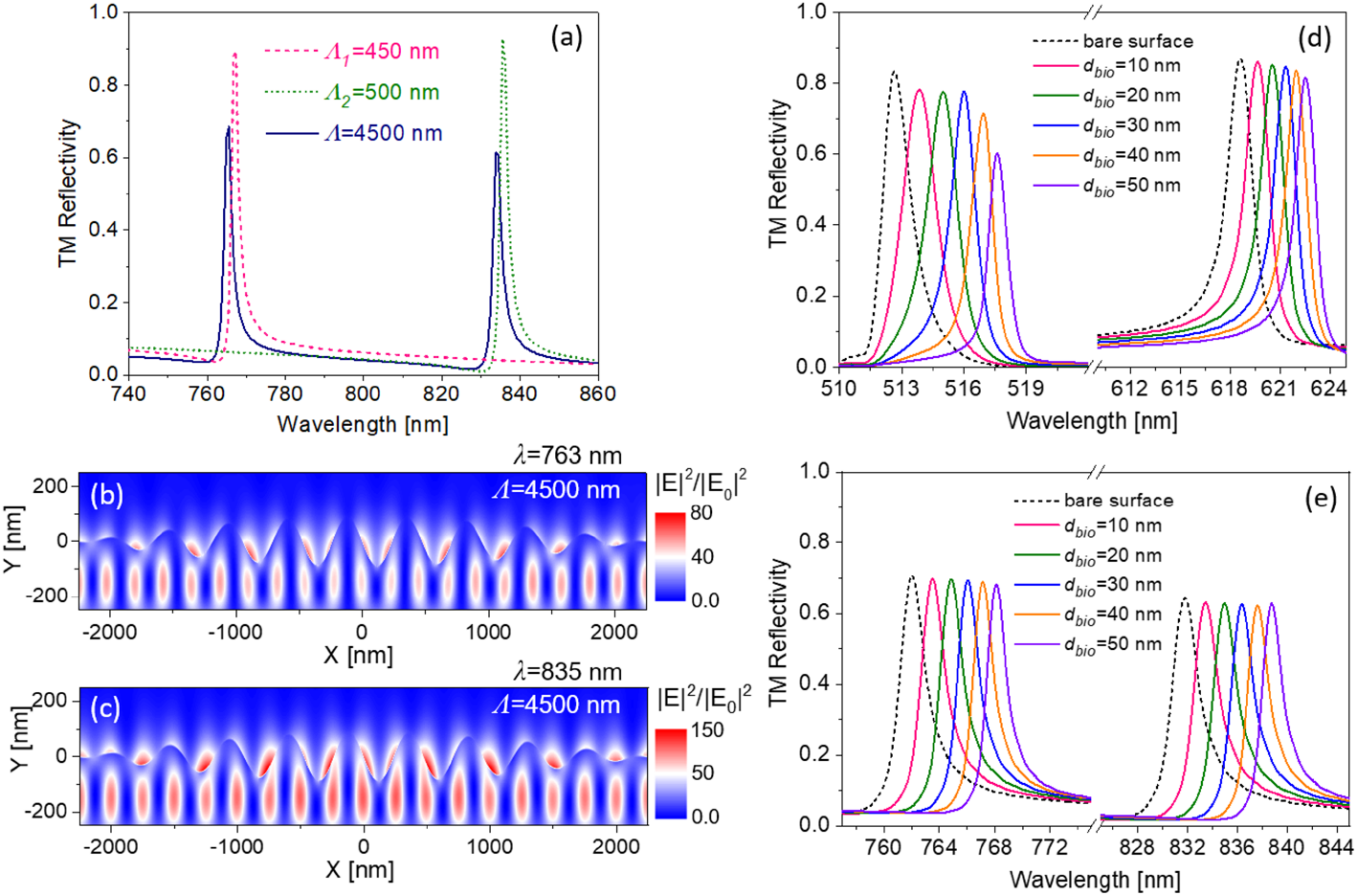
(**a**) TM-polarized reflectivity spectrum as a function of wavelength (*λ*) for the biperiodic waveguide structure with a superimposed period of *Λ* = 4500 nm. (**b**,**c**) Normalized electric field intensity profiles above and within such a waveguide with *Λ* = 4500 nm at two distinct resonant wavelengths. TM-polarized reflectivity curves for (**d**) the single-period grating (*Λ* = 350 nm) with two independent (leaky mode and waveguide mode) resonances, and the same for (**e**) biperiodic structure (*Λ* = 4500 nm) with two independent resonances due to leaky modes.

Alternative to leaky waveguide-gratings with lateral dimensions^16^, binary sequences^17^ and quasi-guided modes^18^, another diffractive architecture that supports dual resonant holograms is the multispectral plasmonic supercell, described previously by us in^19^. Surface development along with field confinement for this bidiffractive structure is shown in Fig. 2b,c. The surface profile of the relief grating is a function of *x*

3 $$f\left( x \right) = a_{1} \sin \left( {\frac{2\pi x}{{\Lambda_{1} }}} \right) + a_{2} \sin \left( {\frac{2\pi x}{{\Lambda_{2} }}} \right),$$

where *Λ*_1_ and *Λ*_2_ are two different periods for each sinusoidal function, while *a*_1_ and *a*_2_ are the amplitude of each sinusoidal function. For simplicity, we consider equal amplitudes for both functions. Each sinusoidal function will have a peak resonance in its reflectivity spectrum. However, by using the combination of two functions, a periodic function is formed that is expected to have two peak resonances. The period of *f* = *(x)* can be found by calculating the Least Common Multiple (LCM) of *Λ*_1_ and *Λ*_2_. To avoid the excitation of leaky modes in these structures, the periods for the sinusoidal function to form *f* = *(x)* were chosen to be *Λ*_1_ = 450 nm and *Λ*_2_ = 500 nm.

Simulation results for each of these sinusoidal functions and their combination are shown in Fig. 2a. The results show that the periodic structure, which has a period of 4500 nm, has two distinctive resonance peaks in the reflectivity curve. The electric field distribution profile for each resonance peak of the periodic structure shows that, although a part of the field power is concentrated in the sensing-layer, the modes related to both of these resonances are waveguide modes in the Si_3_N_4_ diffraction grating layer (Fig. 2b,c). Like for the leaky structure introduced before, the amplitude and periods of the bimodal grating can similarly be optimized to obtain the best coupling efficiency.

## Analysis and sensitivity calculations

The principles in designing the structures described above can be used in biosensing platforms to detect subtle changes in the refractive index of analyte in the biolayer. Adding the biological analyte on top of the waveguide structures will effectively form a new layer. The reflectivity spectrum and hence, the described waveguide modes will be affected by the refractive index of analyte and the thickness of the biolayer. Thus, recording the reflectivity spectra on defined grating structures can in principle be used to determine the refractive index of biological analyte if the layer thickness is controlled.

Calculations showing how a change of thickness of the biolayer will affect the respective resonances, for both the single-period (i.e. leaky) and biperiodic (i.e. waveguide) structures, is shown in Fig. 2d,e. The data shows that *increasing* the thickness of the biolayer will *red-shift* resonance wavelengths of the reflectivity spectrum. Interestingly, the change in resonance wavelength with respect to a change in thickness is not the same for the two different geometries. Specifically, the single period grating is more sensitive to a thickness change than the biperiodic grating as shown in Fig. 2d,e. The first resonance is notably more sensitive to changes in *d*_*bio*_ for the biperiodic structure (Fig. 2e), in which both modes are waveguide modes. This suggests that for cases where *d*_*bio*_ are less well known (as may be the case for less stringent practical applications), a longer wavelength resonance is superior for estimating the refractive index. It also follows that due to this distinct thickness-scaling of the two resonances, a single spectral measurement encompassing both resonances can allow one to extract *d*_*bio*_ as well as its refractive index.

To illustrate the efficiency of these structures for biosensing application, we calculate the surface sensitivity i.e. *dn/dt* to changes in the Refractive Index Unit (RIU) for *d*_*bio*_ = 50 nm (see Table 1). The values in this table have been calculated by assuming a spectral resolution of 0.01 nm, which is considered reasonable for a modern off-the shelf compact single grating spectrometer. As shown for both single-period and biperiodic grating, the sensitivity will be different for the short and long wavelength resonances. Furthermore, long wavelengths are associated with a lower sensitivity for the case of the single-period holograms, while biperiodic holograms provide higher sensitivity. The overall sensitivity of fitting both modes is also seen to be significantly better for the biperiodic holograms. However, the situation appears to be reversed for the sensitivity to the thickness, with both the leaky and waveguide mode of the periodic hologram showing a significantly better sensitivity (independently and when taken together) to that of the dual-resonant hologram. As such one can envision that the biperiodic hologram may find potential applications where *d*_*bio*_ needs to be determined and cannot be inferred a priori or measured using other techniques, particularly for high-throughput analysis^20,21^.

**Table 1:** Modal surface and bulk sensitivities of the structures.

Structure	Optical mode	Surface sensitivity [*RIU*]	Surface sensitivity *Δλ/d*_*bio*_	Bulk sensitivity [*nm/RIU*]
Periodic hologram *Λ* = 350 nm	Leaky *λ* = 510 nm	0.033	0.102	30.129
	Waveguide *λ* = 620 nm	0.043	0.079	23.21
Biperiodic hologram *Λ* = 4500 nm	Waveguide *λ* = 763 nm	0.028	0.12	35.5
	Waveguide *λ* = 835 nm	0.024	0.139	41.3

## Conclusions

Through simulations and calculations based on diffraction grating and Helmholtz wave equations, we have studied two different types of holograms that each support two resonant modes: leaky and waveguide modes in a periodic structure, and two distinct waveguide modes in a biperiodic structure. The performance of these diffractive resonant structures is assessed specifically for potential biosensing applications in aqueous medium. A single-period grating can represent a leaky mode excited at a lower resonant wavelength than its waveguide mode. Though, over a sufficiently broad wavelength window it will be possible to make use of both leaky and waveguide modes of such grating structure. Due to the interferometric nature of the resonances (i.e. multiple resonances in a given wavelength range), the excited modes are effectively narrower and exhibit a higher finesse, and in turn allow for a higher precision. Localizing multiple peaks simultaneously in itself results in a higher precision of localization than a single peak. Our models showed a refractive index sensitivity of 0.033 and 0.043 RIU for leaky and waveguide modes in the single-period grating. The sensitivity to *d*_*bio*_ for this structure was found to be 0.102 and 0.079 for the two modes. The sensitivity is then compared to a superimposed bidiffractive grating structure that incorporates two independent resonances with two encoded periods. In result, a refractive index sensitivity of 0.028 and 0.024 RIU for the first and the second waveguide modes. The sensitivity to *d*_*bio*_ for this bimodal structure was estimated to be 0.12 and 0.139 for both modes. These findings reveal that the leaky-mode waveguide structures, while having a lower refractive index sensitivity, have more than an order of magnitude higher thickness sensitivity than the dual-resonant structure supporting only waveguide modes. This considers the fitting modal sensitivity of both the short and long wavelength resonances for each case. We expect this latter property will have beneficial applications for biosensing, especially where *d*_*bio*_ cannot be accurately controlled or measured.
